# Multi-Focus Imaging and U-Net Segmentation for Mesoscale Asphalt Film Structure Analysis—Method for Characterizing Asphalt Film Structures in RAP

**DOI:** 10.3390/ma18184363

**Published:** 2025-09-18

**Authors:** Ying Wang, Shuming Li, Weina She, Yichen Cai, Hongchao Zhang

**Affiliations:** 1 Key Laboratory of Road and Traffic Engineering, Ministry of Education, Tongji University, Shanghai 201804, China; 2 College of Transportation Engineering, Tongji University, Shanghai 201804, China; lilylsm@163.com

**Keywords:** U-Net segmentation, sub-pixel particle recognition, computer-aided material characterization

## Abstract

This study presents a high-fidelity image acquisition method for asphalt film structure to address the challenge of capturing mesoscale structures, especially fine mineral filler and asphalt mastic. The method is particularly applied to the analysis of the mortar structure in reclaimed asphalt pavement (RAP) mixtures. A digital camera combined with image stacking and texture suppression techniques was used to develop a reproducible imaging protocol. The resulting sub-pixel images significantly improved clarity and structural integrity, particularly for particles smaller than 0.075 mm. U-Net-based segmentation identified 588,513 aggregate particles—34 times more than in standard images (17,428). Among them, 95% were smaller than 0.075 mm compared to just 45% in standard images. Furthermore, segmentation accuracy reached 99.3% in high-resolution images, surpassing the 98.1% in standard images. These results confirm the method’s strong capability to preserve microscale features and enhance fine particle recognition, making it more effective than conventional imaging approaches. This study bridges physical and digital workflows in asphalt material analysis, offering a scalable, reproducible pipeline for fine-structure identification. The methodology provides foundational support for data-driven pavement modeling, material optimization, and future integration into digital twin frameworks for intelligent infrastructure systems.

## 1. Introduction

To realize the concept of green and sustainable construction, the reutilization of reclaimed asphalt pavement (RAP) in road engineering has become a research hotspot. Numerous studies have demonstrated that the characteristics of asphalt mortar in RAP mixtures exert a significant influence on their mechanical and service performance [1,2]. Image-based analysis methods for asphalt film structures provide an essential technical approach for investigating the spatial distribution of asphalt mortar [3]. Accurate characterization of the microstructural features of asphalt mortar within RAP mixtures is therefore critical for gaining deeper insights into their behavior and for improving overall performance.

Reconstructing multiscale coupled digital models of asphalt film structures based on digital imaging—spanning micro-, meso-, and macroscales—is critical to developing an integrated framework for performance prediction and multiscale optimization in material design [4,5]. Within this context, the accurate digital representation of the meso-structure remains a persistent and unresolved challenge [6,7]. Recent studies have employed digital images to inform the reconstruction of the Cohesive Zone Model (CZM), capturing the interfacial behavior between aggregates and asphalt mastic [8,9,10,11]. These models are central to linking macroscopic mechanical performance with underlying meso-structural features. The CZM framework reflects the composite meso-structure composed of asphalt binder, mineral filler, and fine aggregates. When combined with aggregate packing models, CZMs enable simulation of the global mechanical response of asphalt mixtures. However, due to limitations in current observational techniques, the anisotropic behavior of asphalt mixtures can often be reproduced only at the macroscale. To model features such as asphalt mastic, asphalt mortar, and interfacial zones between aggregates and binder, many studies adopt the Representative Volume Element (RVE) [12,13,14], simplifying the heterogeneous structure into equivalent isotropic materials to approximate macroscale responses. While homogenized models provide valuable insights, the inherent anisotropy of asphalt mixtures [15,16,17,18] remains a significant challenge. To accurately reconstruct meso-structures and preserve their anisotropic characteristics, recent approaches rely heavily on high-resolution imaging from CT scanning and 2D digital cameras [19,20,21]. The fidelity of model reconstruction is highly dependent on image resolution, which governs the completeness and precision of the internal structure representation. Unlike metals or polymers, asphalt mixtures are dense, opaque, and non-conductive, making them difficult to sample and visualize on small scales. Additionally, strong size effects within the material complicate efforts to capture representative structural features using small specimens. Consequently, CT imaging and high-resolution 2D imaging remain the primary modalities for image-based modeling of asphalt mixtures [22,23]. These techniques reliably identify aggregate particles larger than 2.36 mm [22,23,24,25,26,27] and, in some cases, down to 0.2 mm [28,29,30]. The ability to accurately delineate particle boundaries [31] is essential for reliable sampling and segmentation. A complete digital reconstruction of asphalt mixtures must incorporate precise meso-structural modeling that reflects the material’s inherent heterogeneity. Cutting-edge research is now pushing the limits of imaging resolution to achieve submicron 3D reconstructions of meso-structures [32,33]. Attaining sub-pixel precision requires not only advances in imaging hardware but also the development of efficient, adaptive image acquisition workflows and intelligent image processing systems. Integration of state-of-the-art image processing algorithms is essential for generating high-quality standardized images, optimized for deep learning-based segmentation and sampling. During the image digital processing (IDP) phase, it is critical to enhance image resolution to the sub-pixel level [34,35,36,37], thereby enabling precise characterization of meso-scale features and facilitating multiscale intelligent modeling of asphalt mixtures.

In asphalt mixtures, the asphalt film structure—defined in this study as the Interfacial Transition Zone (ITZ) between the asphalt binder and aggregates—constitutes a representative and critical mesoscale feature. This region exhibits a complex multiphase composition, incorporating a dense distribution of mineral particles typically ranging from 0.1 μm to 100 μm. Accurate reconstruction of this structure necessitates high-resolution modeling capable of capturing the fine-scale heterogeneity and spatial variability of constituent materials. Previous studies have established that the gradation of mesoscale particles has a direct impact on the macroscopic mechanical behavior of asphalt mixtures [38,39,40,41]. When appropriately graded, these particles may contribute to the formation of a load-bearing skeleton that significantly enhances structural integrity [42]. However, the limited resolution of available imaging techniques has posed substantial barriers to the validation of such findings, particularly in the case of fine-scale structures. The concept of asphalt film—encompassing both isolating and oily films—was first introduced by Campen in 1959 [43], who, through theoretical derivations and assumptions, proposed an ideal film thickness of approximately 6–8 μm. Although many researchers have since elaborated on Campen’s theoretical model [44,45,46,47], the absence of high-fidelity digital imaging has precluded direct experimental verification, leaving critical aspects of the asphalt film structure largely inferred rather than observed.

This study presents a high-resolution image acquisition and processing framework for asphalt mixtures, integrating a U-Net-based deep neural network to systematically address the challenges. The proposed method leverages micron-level resolution imaging devices in combination with advanced image processing software and tailored U-Net architecture. A standardized set of procedures and a comprehensive processing pipeline have been developed to ensure consistency and reproducibility. Through this approach, high-fidelity digital images of asphalt mixtures can be obtained with reliable sub-pixel accuracy, thereby providing a robust data foundation for comprehensive digital representation and intelligent analysis of asphalt mixture microstructures.

## 2. Methodology

### 2.1. Image Acquisition Equipment

From the perspective of digital image acquisition, existing techniques for capturing internal structural information of materials can be broadly categorized into two classes. The first category comprises transmission-based methods, such as ultrasonic testing and industrial computed tomography (CT), which require specific intrinsic properties of the material, such as electrical conductivity [48]. These techniques rely on the attenuation or transformation of energy beams as they penetrate the material and are governed by well-established testing standards and protocols. They enable effective detection of internal microstructural features with high precision and depth. The second category includes surface-based imaging techniques, which are less dependent on the material’s physical properties. These methods—such as optical microscopy, surface ray inspection, eddy current testing, magnetic particle testing, and microwave detection—primarily capture the reflection or scattering signals emitted from the material surface. While they often require high-quality sample preparation, they can produce high-resolution, sharp grayscale images with relatively low contrast. Among these approaches, digital cameras have emerged as versatile imaging tools capable of acquiring high-resolution and high-contrast color images without relying on specific physical attributes of the specimen. Their broad applicability makes them an attractive option for capturing surface structural features. However, the absence of standardized acquisition and processing workflows limits the extent to which these images can meet the stringent sub-pixel accuracy requirements associated with asphalt mixture structural analysis.

This section provides a systematic comparison of three representative imaging techniques—industrial CT, scanning electron microscopy (SEM), and digital cameras—in terms of their capability and applicability for extracting internal structural information from asphalt mixtures.

#### 2.1.1. Industrial CT

Industrial computed tomography (CT) is one of the most widely adopted imaging techniques in current asphalt mixture research. It enables the non-destructive acquisition of 3D structural images at the macroscale, preserving the integrity of the specimen throughout the scanning process. Industrial CT imposes minimal constraints on sample size and physical properties, making it well-suited for a broad range of applications involving internal structural characterization of asphalt mixtures.

**Figure 1:**
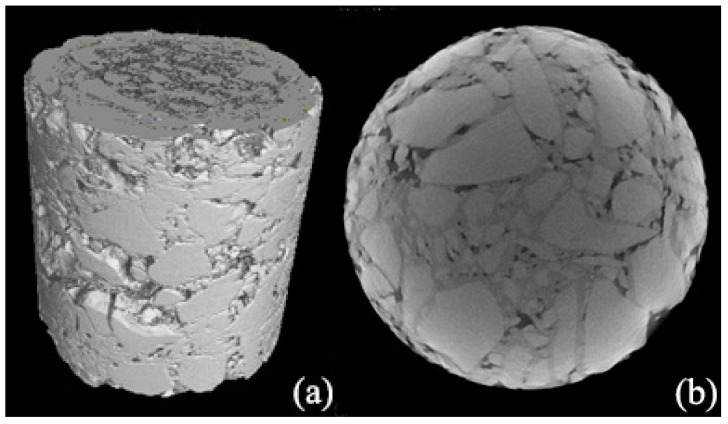
CT image illustration of asphalt mixture. (**a**) 3D view; (**b**) X-Y plane view.

As a typical non-destructive transmission-based imaging technique, industrial computed tomography (CT) can capture high-resolution structural images of asphalt mixture specimens beneath their surfaces. However, its imaging precision is limited to the macroscale, resulting in relatively low contrast and sharpness [49,50,51]. Additionally, due to the beam hardening effect, 3D images often exhibit brightness non-uniformity, characterized by enhanced edge brightness and reduced central brightness. This phenomenon significantly complicates subsequent digital image processing (DIP) and indirectly reduces the image processing quality and spatial resolution. The image data presented in Figure 1 were acquired using an XT H225 ST micro-CT system (Nikon Corp., Minato-ku, Tokyo, Japan), which exhibits the beam hardening effect. To mitigate this artifact, several researchers in previous studies have attempted to scan smaller asphalt mixture specimens. However, this approach contradicts the fundamental design principle of industrial CT, which aims to preserve the structural integrity of an entire specimen.

The imaging accuracy of commonly used industrial CT equipment typically does not exceed 0.5 mm [18,27]. Although the theoretical resolution of CT scanning can reach 0.5 mm, practical structural features are often discernible only at sizes much larger than this theoretical value due to factors such as beam hardening effects and imaging noise. Due to limitations in the imaging capabilities of industrial CT during the acquisition phase, studies involving asphalt mixture model reconstruction based on CT images typically only replicate macroscopic structural features. Consequently, the construction and characterization of multiscale digital models spanning the mesoscopic to macroscopic range remains challenging.

#### 2.1.2. Scanning Electron Microscopy (SEM)

**Figure 2:**
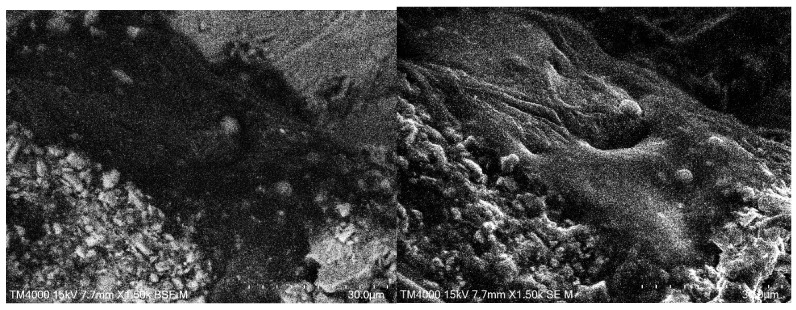
Comparison of SEM BSE (**left**) and SE (**right**) images.

Scanning electron microscopy (SEM) is a widely employed technique for acquiring high-resolution images of material surfaces. SEM-generated digital images typically exhibit superior contrast and sharpness, rendering the method highly suitable for analyzing microscale structural features in various materials. However, due to its specific imaging principles, SEM requires samples to undergo stringent specimen preparation procedures, or the material itself must possess favorable physical properties such as electrical conductivity or optical transparency—conditions that are difficult to meet with asphalt mixture specimens. Asphalt mixtures exhibit poor conductivity, high mass and density, low optical transparency, and considerable hardness, making them unsuitable for conventional surface morphology observation techniques commonly used in materials science. Despite these challenges, the exploratory investigations in this study included SEM imaging of fragmented asphalt concrete particles. The resulting images, as shown in Figure 2, revealed additional complexities associated with characterizing the internal structures of asphalt mixtures.

To minimize disturbance to the microstructural integrity of asphalt mixtures, this study employed a freeze–fracture technique to prepare samples for SEM imaging. Compacted asphalt mixture specimens were frozen at −18 °C for 24 h and subsequently fractured. Fragments smaller than 30 mm that exhibited relatively flat fracture surfaces containing visible aggregate particles were selected for SEM observation. The images were obtained using a TM4000Plus benchtop SEM (Hitachi High-Tech Ltd., Ibaraki-ken, Tokyo, Japan). The corresponding backscattered electron (BSE) and secondary electron (SE) images are shown in Figure 2. The BSE image primarily reflects the two-dimensional distribution of distinct material phases, while the SE image reveals localized three-dimensional surface morphology at the scan site. As observed in Figure 2, mineral filler particles that appear to lie on the same plane in the BSE image are situated at different depths in the SE image. This discrepancy indicates that neither BSE nor SE imaging can reliably capture the spatial distribution of mesoscale aggregate particles within the asphalt film structure. Consequently, SEM imaging is unsuitable for investigating particle dispersion within the asphalt film structure of asphalt mixtures.

As SEM operates at nanoscale resolution, conventional mechanical sectioning methods can introduce significant artifacts during sample preparation. The heat generated during cutting may locally soften the asphalt binder, resulting in the displacement of aggregate particles and thereby compromising the integrity of the microstructural features. Such thermal-induced disturbances severely distort the statistical representation of internal structural characteristics. Therefore, relying solely on the preparation of flat observation surfaces is insufficient to overcome the fundamental limitations of SEM in the mesoscale structural analysis of asphalt mixtures.

#### 2.1.3. Digital Camera

Digital cameras represent one of the most widely adopted imaging tools in the characterization of current materials. Driven by rapid commercial advancements, these systems have demonstrated substantial improvements in image acquisition accuracy and operational convenience. Most state-of-the-art digital camera systems available on the market can capture digital images with micrometer-level precision. Among these devices, the type of image sensor serves as a critical distinguishing factor. Charge-coupled devices (CCD) and complementary metal–oxide semiconductor (CMOS) sensors are the core imaging components in high-resolution systems commonly used in materials research. The selection between CCD and CMOS sensors for capturing mesoscale features of asphalt mixtures depends largely on the specific requirements of subsequent image processing tasks.

Compared to CCD sensors, CMOS sensors are more cost-effective, exhibit superior radiation resistance, and are applicable across a broader range of imaging scenarios, contributing to their more rapid technological development. Although CMOS sensors traditionally suffer from higher noise levels under equivalent imaging precision, advancements in Active Pixel Sensor (APS) technology have substantially mitigated fixed-pattern noise and other structural artifacts [52]. In the context of asphalt film structure analysis, which requires the acquisition of large volumes of high-resolution digital images for statistical evaluation, the selection of CMOS sensors represents a more practical choice. This decision is based on a balanced consideration of time efficiency and economic feasibility, making CMOS the preferred image acquisition component for the methodology presented in this study.

### 2.2. Specimen Preparation Requirements

The original gradation of the RAP mixture used in this study and the gradation of the pure aggregate particles after ignition treatment are presented in Table 1.

To enhance the formation of interfacial structures, natural aggregate particles within the 26.5–13.2 mm range were incorporated, resulting in the mixture gradation shown in Table 2. An asphalt binder with fundamental properties, summarized in Table 3, was also incorporated in this study to supplement the binder content, thereby improving workability and facilitating compaction. The fundamental properties of the asphalt mixtures used in this study are summarized in Table 4.

The sampled specimens consisted of flat cross-sections obtained by cutting asphalt mixtures after compaction (as shown in Figure 3①). To prevent image distortion caused by factors such as stretching [53], which could compromise the accuracy of digital images, it is essential to ensure that the cross-sections of the asphalt mixture are as flat as possible. This minimizes the risk of curvature or excessive roughness on the surface, which could negatively impact the precision of the image data.

### 2.3. Image Acquisition Platform

The experimental setup employs a Canon EOS 90D (18–135 mm) USM kit lens equipped with a Canon EF 100 mm f/2.8 USM macro lens (Canon Inc., Zhongshan, China) (Figure 3②). The field of view is 24 × 36 mm^2^, with a focal distance of 0.31 m and a minimum focusing distance of 0.14 m. To ensure proper exposure for each photograph, the aperture is set to F2.8, achieving optimal brightness without using continuous exposure shooting. The ISO is automatically adjusted to avoid noise sharpening caused by excessively high ISO values. To enhance image quality, a bar lighting system (Figure 3④) was incorporated into the setup to ensure even illumination across the specimen cross-section. Additionally, a combination scale (Figure 3③) was used to measure the flatness of the X–Y–Z three-dimensional translation stage (Figure 3⑤), adjusting the distance between the asphalt mixture cross-section and the digital camera lens. The X–Y–Z three-dimensional translation stage is utilized to stabilize the digital camera and control the range of the specimen surface within the camera’s field of view, ensuring that the distance and parallelism between the camera and the specimen cross-section are optimized, thereby maximizing imaging precision.

**Figure 3:**
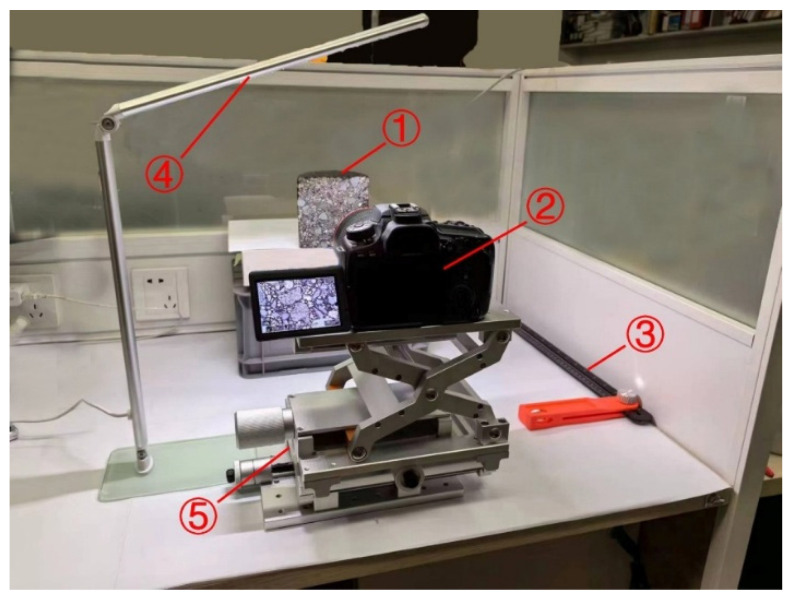
Digital image acquisition operation platform. ① Canon EOS 90D with an 18–135 mm USM kit lens. ② Canon EF 100 mm f/2.8 USM macro lens. ③ Combination scale for flatness measurement of the translation stage. ④ Bar lighting system providing uniform illumination of the specimen cross-section. ⑤ X–Y–Z three-dimensional translation stage for stabilizing the digital camera and controlling the specimen position.

Based on the objectives and in combination with the sampling equipment and auxiliary tools, this study developed a digital image sampling experimental platform, as shown in Figure 3. This surface image acquisition method demonstrates significant advantages in meeting the comprehensive digitalization requirements for asphalt mixture research. The standardized image acquisition and processing workflow facilitates the advancement and expansion of related research in the digitalization of asphalt mixtures. The experimental method for acquiring and processing surface digital images of asphalt mixtures, as described in this section, was conducted under ambient temperature conditions (25 ± 1 °C).

### 2.4. Photography Methodology

#### 2.4.1. Platform Setup

To ensure that the designed acquisition method can capture complete high-resolution images as expected and is repeatable, it is necessary to supplement the specific details and reasons behind each operational step.

(1) Leveling of the Surface

To reduce the number of individual images needed for image acquisition, alleviate the difficulty of subsequent stitching and merging, and shorten processing time, it is essential to minimize the potential for distortion by ensuring that all planes are parallel to each other. This ensures the efficiency of the image acquisition process. In this method, the experimenter must level all surfaces used during the imaging process, including the operating table, the X–Y–Z three-dimensional stage, and any supporting blocks, using calibrated spirit levels prior to the experiment.

(2) Instrument Positioning

To ensure that each pixel within the field of view represents the smallest actual area that is in focus, the object being photographed must be positioned at the focus of the lens, i.e., at the minimum focusing distance (14 cm). The arrangement of the imaging equipment and the scene setup is designed to ensure that the optical axis of the macro camera lens is perpendicular to the flat surface of the asphalt mixture. This helps control perspective and reduce the occurrence of perspective distortion.

(3) Lighting Setup

Adequate lighting conditions are essential for providing a bright field of view, reducing reflections (or glare), and minimizing segmentation errors during image recognition. Therefore, a strip light source should be positioned on the same plane as the flat surface of the asphalt mixture specimen. This ensures that the entire plane is illuminated, minimizing specular reflections.

By rigorously adhering to steps (1)–(3) in preparation for image acquisition, the accuracy and completeness of the captured images can be controlled, providing a reliable foundation for subsequent analysis and research based on high-resolution images.

#### 2.4.2. Multi-Focus Imaging Protocol

In this experimental method, a multi-focal-plane acquisition protocol was developed to enhance the depth of field in high-resolution imaging of asphalt mixture cross-sections. Each field of view is captured at nine distinct focal planes. These planes are arranged in a bidirectional tri-grid pattern to ensure sharp imaging of all mineral particles within the specified flatness tolerance. The post-acquisition process follows a two-stage stacking and stitching procedure. First, intra-field focus stacking is carried out using Adobe Photoshop’s Auto-Align and Auto-Blend functions to generate locally uniform high-definition images. Second, adjacent stacked fields are merged with a 40–50% overlap to produce seamless, high-resolution composites. This procedure achieves sub-pixel resolution across large specimen areas while preserving geometric and radiometric fidelity. As a result, it establishes a reliable basis for meso-structural modeling and machine learning-based feature extraction.

In this experimental method, a highly displacement-sensitive digital camera is employed. To prevent incomplete images from being acquired during the operational process—due to certain actions that may cause misalignment during subsequent stitching and lead to erroneous information in the final images—several critical operational details are outlined here. The image acquisition method designed in this study does not employ any special color or tone modes. The shutter action must be swift and precise, with consistent pressure and amplitude maintained during the pressing process, to avoid misalignment of the captured field of view or uneven exposure, which could lead to noise and distortion.

**Figure 4:**
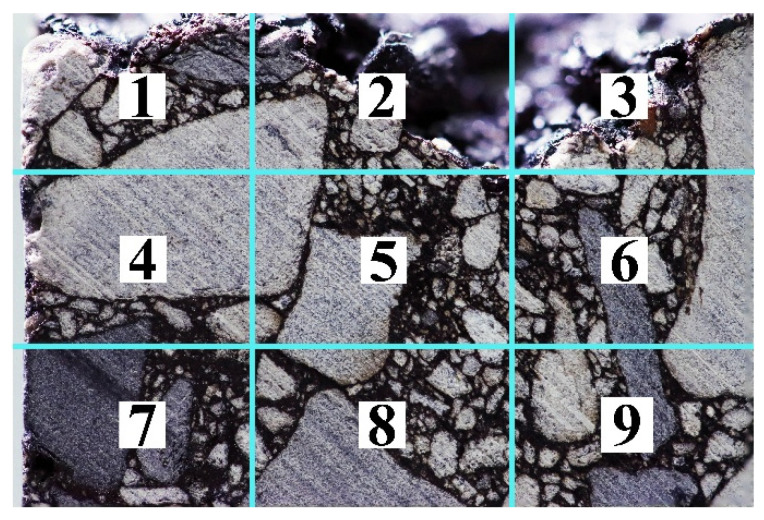
Illustration of bidirectional trisection reference lines in the monitor.

To achieve precise positioning, this method utilizes the manual focusing function of the digital camera. Throughout the process, the positioning of the sample is monitored in real time on the monitor to ensure accuracy, while the focal point is manually set. To ensure that high-resolution images are obtained with equal clarity at each position during subsequent stitching, each field of view is divided into a bi-directional, three-part reference grid (as shown in Figure 4), with the center of each rectangle used as the focus point for capturing images. After performing this procedure, each field of view corresponds to nine images captured at different focal points. After each field of view is captured, the position of the asphalt mixture sample and associated equipment remains unchanged, and the three-dimensional movable platform is adjusted either horizontally or vertically. The digital camera monitor is then used to modify the area being captured, ensuring that each new range overlaps with the previous range by 40% to 50%. This overlap is required for the stacking function in Photoshop. During the movement process, constant attention must be paid to the position of the target image to ensure that the overlapping requirement is met for the next field of view. The three-dimensional movable platform should follow the principle of “small adjustments, multiple times” during the adjustment process to prevent large shifts that may cause the target image to fall outside of the view.

## 3. Digital Image Processing (DIP) Workflow

To improve image accuracy and enhance the completeness and precision of the mineral aggregate structure information presented within the asphalt mixture, the digital image processing (DIP) method introduced in this paper uses Photoshop CC 2019. This method restores sub-pixel level aggregate particle details through stacking and stamping techniques.

### 3.1. Multi-Focus Stacking and Stitching

To obtain a complete cross-sectional image of the asphalt mixture with sub-pixel accuracy, it is necessary to stitch and merge images captured at different focal points within the same field of view, as well as local digital images from different fields of view. Isolated and scattered local image data is insufficient to support systematic research. In the image stitching and merging process, Photoshop’s stacking function is widely used in research domains that require high fidelity and precision due to its superior edge alignment, color consistency, and detail preservation capabilities [54,55,56,57], such as star trail observation and biological specimen preservation [57,58,59,60]. This study employs stacking to achieve lossless, sub-pixel accurate image stitching and composition. The stacking method demonstrates high robustness and is well-suited for processing high-resolution images with complex details, such as the cross-section of asphalt mixtures. The DIP procedure proposed in this study consists of two stacking steps based on the type of image being processed:

(1) First Stacking: Merging Images with Different Focal Points within the Same Field of View

**Figure 5:**
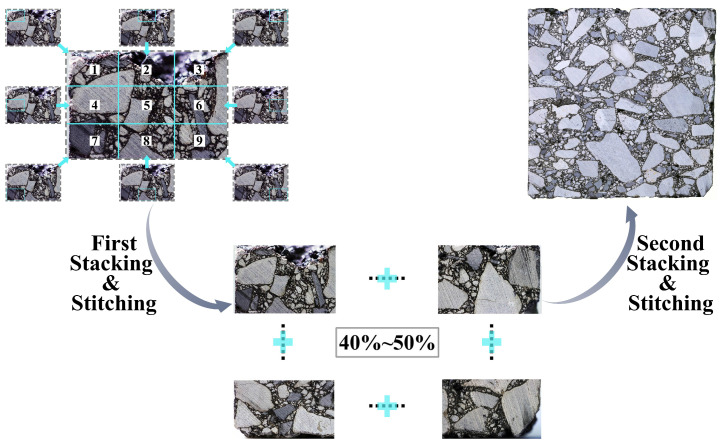
Flowchart of the image stitching and merging process.

As described in Section 2.4.2, nine cross-sectional images with varying focal points were collected for each captured field of view (see Figure 4). According to the workflow shown in Figure 5, Photoshop’s stacking function was applied to merge these nine images within the same field, resulting in a single Focus Stacked Image with uniform sharpness.

(2) Second Stacking: Merging Images from Different Fields of View

Although the first step provides Focus Stacked Images for each localized region, each field of view covers only a small portion of the structural area, and the overall information completeness remains limited. To fully capture the internal structural characteristics of the asphalt mixture, it is necessary to perform a second round of image stitching across different fields of view.

Following the workflow illustrated in Figure 5, this two-step stacking procedure achieves seamless stitching of images from adjacent fields, ultimately generating a high-resolution, information-rich digital cross-sectional image of the asphalt mixture for subsequent structural and performance analyses.

**Figure 6:**
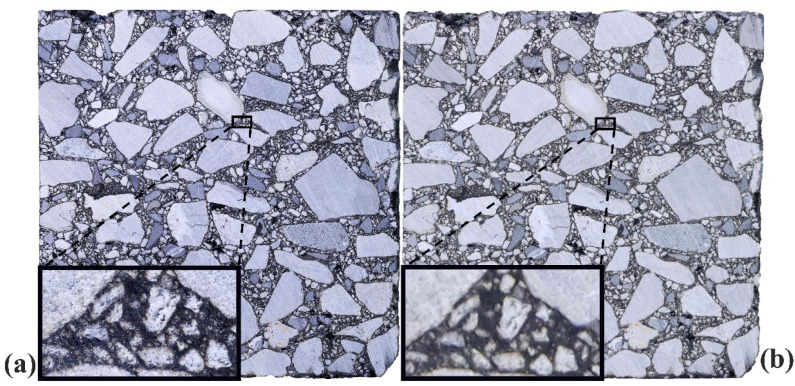
Image comparison of the same asphalt mixture cross-section. (**a**) High-Resolution Image; (**b**) Standard Image.

**Figure 7:**
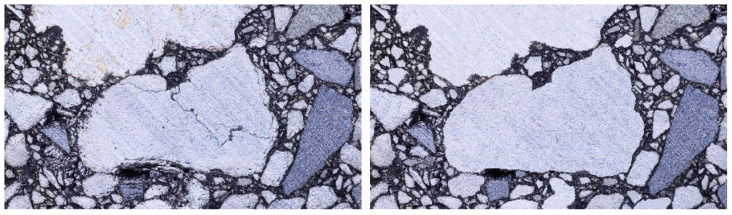
Comparison of texture removal effects ((**Left**): Local region of High-Resolution Image; (**Right**): Local region of Standard Image).

The High-Resolution Image obtained through the aforementioned acquisition and processing workflow preserves the overall geometry and aspect ratio consistent with the directly captured Standard Image, while retaining significantly more mesoscale structural details, as illustrated in Figure 6. A comparative analysis between Figure 6a and Figure 7b demonstrates that the High-Resolution Image, generated via the proposed method, exhibits comparable morphological consistency and external contour integrity with the Standard Image, without any evident information loss. Moreover, local magnification reveals clearer aggregate boundaries and additional mesoscale structural features. The comparison results in Figure 6 indicate that this high-resolution image acquisition and processing method not only ensures the accuracy of the structural information but also enhances image resolution to the sub-pixel level. This advancement provides a critical foundation for intelligent recognition, image segmentation, and statistical analysis in the digital investigation of asphalt mixtures.

### 3.2. Texture Suppression

While the High-Resolution Image obtained through the stacking-based method effectively preserves the mesoscale structural information of the asphalt mixture, it also retains a number of redundant features, such as aggregate surface textures, fragmented fine particles generated during specimen preparation, and asphalt residues adhering to aggregate surfaces due to thermal effects during cutting (as shown in Figure 7). These local features do not contribute to accurate particle size distribution analysis and may introduce misclassifications during subsequent aggregate identification and segmentation based on deep neural networks.

To ensure the accurate representation of image features, a texture suppression process was introduced during the image processing phase to eliminate non-structural information irrelevant to aggregate identification from the High-Resolution Image. Given that particle size distribution is derived from the statistical analysis of equivalent particle diameters and that fragmented particle segments can significantly distort the results, the proposed method prioritizes the integration of fractured regions into complete particles to minimize estimation errors. Furthermore, to avoid introducing contour deviations during texture suppression, the algorithm strictly preserves the edge pixel locations of each particle, applying smoothing only to the high-frequency information within particle interiors.

Given that the deep neural network algorithm adopted in this study assigns greater weights to low-frequency boundary pixels during training, even minor redundant features—such as natural textures or asphalt residues—may be misclassified as valid boundaries, thereby compromising particle segmentation accuracy. Accordingly, the implementation of standardized texture suppression significantly enhances the compatibility and accuracy of asphalt mixture cross-sectional images for learning and training tasks in deep neural network applications.

The stacked cross-sectional images of asphalt mixtures often contain redundant information, including the surface textures of aggregate particles, fragmented aggregates caused during specimen compaction or cutting, and asphalt residues adhering to the cut surfaces due to thermal effects. While the actual size of fragmented particles is difficult to determine, integrating these fragments into a single complete particle introduces less error in particle size distribution estimation than treating them as multiple smaller particles. This is because particle size distribution is based on the frequency count of particles falling within predefined size ranges. The deep neural network algorithm employed in this study assigns greater weights to low-frequency edge pixels for particle boundary detection. However, redundant features such as natural aggregate textures or asphalt residues on the particle surfaces—also represented by low-frequency pixels—may be misclassified as valid boundaries, leading to segmentation errors during model training. Therefore, during texture suppression, particle edges are strictly preserved to avoid introducing additional boundary deviations and maintain segmentation accuracy.

### 3.3. Image Acquisition and Processing Workflow

This study proposes a standardized and systematic workflow for the acquisition and processing of asphalt mixture cross-sectional images, aiming to provide high-quality data for subsequent meso-structural recognition and digital analysis. Cross-sections of asphalt mixture specimens were obtained by cutting molded samples to produce flat, unwarped surfaces with surface roughness controlled within acceptable limits. High-resolution macro imaging was conducted using a Canon EOS 90D equipped with a 100 mm f/2.8 USM macro lens under standardized lighting and temperature conditions (25 ± 1 °C). To achieve full-area, multi-focus coverage, an X–Y–Z precision motorized stage was employed to adjust the camera-to-sample distance.

To obtain high-resolution images at the sub-pixel level, this study employed a two-stage image stacking process using Adobe Photoshop. First, multi-focus images within a single field of view were fused through focus stacking to generate sharp, detail-preserving images. Subsequently, local focus-stacked images captured at different spatial positions were seamlessly stitched together to reconstruct a complete sub-pixel resolution cross-sectional image of the asphalt mixture. Following image reconstruction, a texture suppression process was applied to eliminate non-structural features introduced during sample preparation, cutting, or molding—such as aggregate surface textures, asphalt residues, and fragmented particles. Throughout this process, aggregate boundary integrity was strictly preserved to enhance the compatibility and accuracy of the images for deep neural network-based segmentation and learning tasks.

**Figure 8:**
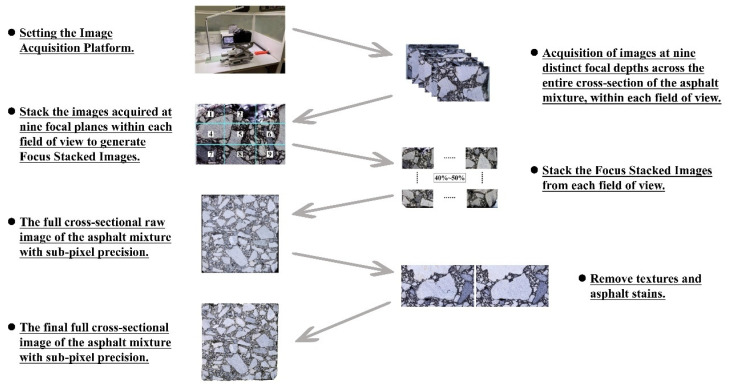
Flowchart of asphalt mixture cross-section image acquisition and processing.

Through the multi-stage, high-resolution image acquisition and processing workflow illustrated in Figure 8, this study successfully achieved high-fidelity capture of the meso-structural characteristics of asphalt mixtures. The resulting digital images exhibit enhanced performance in intelligent recognition, feature extraction, and statistical analysis. This provides a robust technical foundation for the digital multiscale modeling of asphalt mixtures, supporting the development of comprehensive material performance prediction frameworks and multiscale optimization design theories.

Given the irregular morphology and blurry boundaries of fine mineral particles in asphalt mixtures, this study integrates a pixel-weighted boundary enhancement mechanism into the U-Net segmentation framework. Each pixel is assigned a dynamic weight combining class frequency and proximity to the nearest particle boundaries, ensuring stronger boundary recognition during training. Furthermore, skip-connection-enhanced architecture enables precise reconstruction of low-resolution features, and a large-tile small-patch segmentation strategy balances memory usage and segmentation accuracy. Experimental results show that this approach achieves 99.3% segmentation accuracy, significantly outperforming conventional standard image methods and providing reliable data support for meso-scale particle analysis.

## 4. U-Net Segmentation Framework

In the following sections, the digital cross-sectional images of asphalt mixtures obtained through the proposed multi-step method are referred to as high-resolution images, whereas images directly captured from asphalt mixture cross-sections are termed standard images.

Due to the inherent heterogeneity and irregular morphology of aggregate particles within asphalt mixtures, accurately identifying small-sized, irregular-shaped aggregates and mineral fillers remains a significant challenge. To reliably extract the structural characteristics of the effective asphalt film, this study leverages the powerful learning capabilities of the Deep Neural Network (DNN) to capture the boundary information of aggregate particles, which serves as the foundation for subsequent structural feature analyses of the effective asphalt film. DNN learning was first introduced by Geoffrey Hinton in 2006 [60] to address the issue of gradient vanishing, which arises as the computational depth of backpropagation-based neural networks increases. To mitigate gradient vanishing or entrapment in local minima during training, DNNs typically employ a layer-wise training strategy. Common layer-wise trained models include Convolutional Neural Network (CNN) and Fully Convolutional Network (FCN), with the latter specifically designed for end-to-end, pixel-level image segmentation tasks.

**Figure 9:**
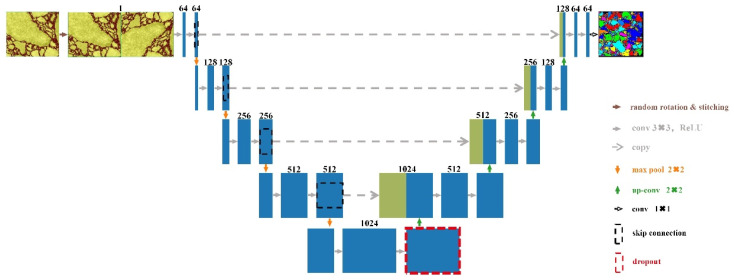
Schematic diagram of U-Net architecture. (The blue and green rectangular blocks represent multi-channel feature maps and copied feature maps, respectively. The numbers at the top of the blocks indicate the number of channels).

Among the various FCN models, U-Net is renowned for its broad applicability and fast computation speed. It is one of the earliest artificial intelligence architectures designed and widely accepted, excelling particularly in complex small-sample learning tasks. U-Net, proposed by Ronneberger [61], serves as the core algorithm of an FCN in the field of biomedical image segmentation. Although it has undergone various modifications to adapt to more specific scenarios, U-Net still maintains a significant advantage in training speed over its variant networks. U-Net preserves the architecture feature of FCN that omits fully connected layers, enabling it to operate on input images of different sizes. The overall structure follows the FCN’s encoder–decoder design, capturing contextual information through the contracting path and performing precise feature localization through the expanding path. Because the architecture resembles the letter “U,” it was named U-Net, as illustrated in Figure 9.

Based on the architectural features of FCN, U-Net implemented several key improvements: (1) Data augmentation was applied to the original dataset to enhance model generalization and prevent overfitting, with Figure 9 demonstrating one technique—random flip-and-stitch. (2) Only valid convolutions were used, combined with skip connections to ensure that the segmentation results contain only fully valid pixels from the input image and can completely recover low-resolution details lost during up-sampling. (3) Weight map calculation was introduced to balance the relationship between pixel quantity and importance, assigning greater weights to less frequent pixels through Equation (1) to address the challenge of distinguishing contacting positions between sampled objects. (4) A design of large tiles with small patches was employed to improve GPU utilization efficiency.

(1) $$\omega_{\left(x\right)}=\omega_{c}\left(x\right)+\omega_{0}\cdot exp\left(-\frac{{\left(d_{1}\left(x\right)+d_{2}\left(x\right)\right)}^{2}}{2\sigma^{2}}\right)$$

$\omega_{\left(x\right)}$—pixel weight value;

$\omega_{c}\left(x\right)$—category weight.

(2) $$\omega_{c}\left(x\right)=\frac{1}{{Freq.}_{c}}$$

${Freq.}_{c}$—frequency of occurrence of Category c pixels;

$\omega_{0}$—the boundary weighting coefficient set to the empirically optimal value of 1;

$d_{1}\left(x\right)$—the shortest Euclidean distance from pixel *x* to the nearest pixel of the boundary of a sampled target;

$d_{2}\left(x\right)$—the Euclidean distance from pixel *x* to the second closest pixel of the boundary of a sampled object;

σ—the boundary influence range set to an empirically optimal value of 5.

U-Net, along with its direct skip connections, enables the training of efficient models even on relatively small datasets, making U-Net particularly well-suited for few-shot learning tasks.

## 5. Result Analysis

### 5.1. Statistical Results

**Figure 10:**
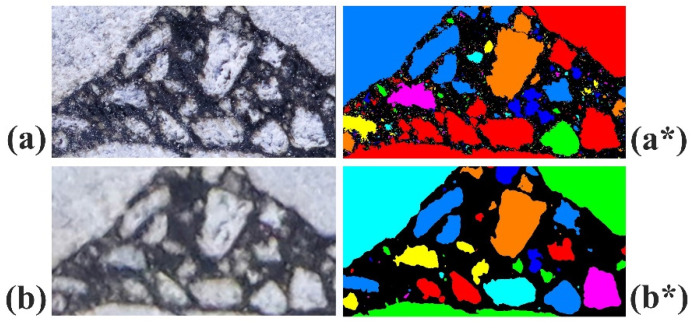
Comparative illustration of local aggregate particle recognition result labels in asphalt mixtures. (**a**,**b**): Enlargements of the local areas indicated in Figure 6. (a*,b*): The respective mineral particle label maps corresponding to (**a**,**b**), obtained through the trained U-Net model.

There is a significant difference in imaging quality between the High-Resolution Image and the Standard Image. Figure 10 shows a comparison of the mineral particle identification results between the High-Resolution Image and the Standard Image. In Figure 10, (a) and (b) display the enlarged local regions shown in Figure 6, while (a*) and (b*) are the mineral particle label maps obtained through the trained U-Net model, corresponding to (a) and (b), respectively. From the comparison between (a) and (b) in Figure 10, it is evident that the High-Resolution Image has higher resolution and clarity compared to the Standard Image. As a result, the generated mineral particle label map displays more complex particle contours and is able to identify more small-particle-sized minerals.

**Figure 11:**
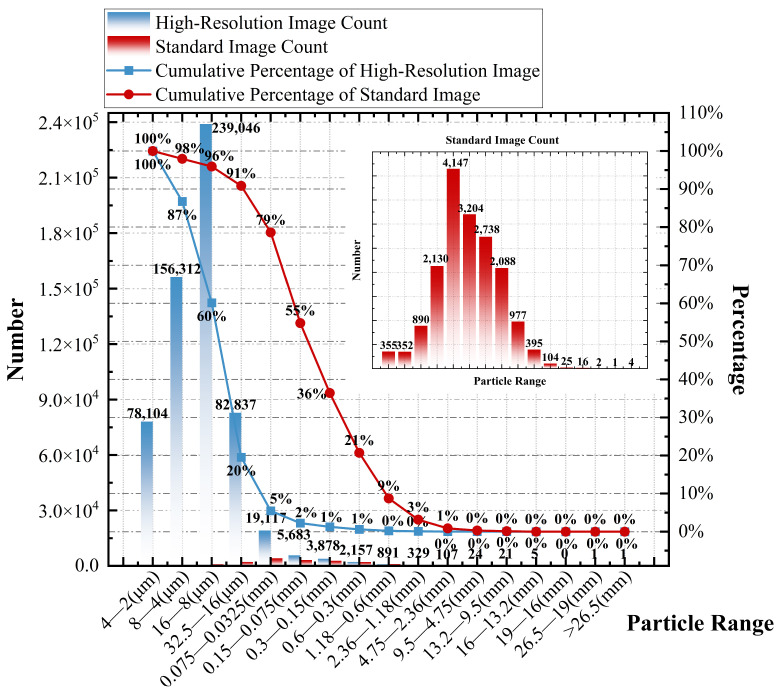
Statistical results of aggregate particles.

The high-resolution asphalt mixture cross-sectional image and the standard asphalt mixture cross-sectional image, after being segmented and labeled by U-Net, were used to statistically determine the frequency distribution of mineral particle size ranges, with particle diameter as the selection criterion. The results are shown in Figure 11.

Figure 11 shows the particle size distribution statistics of mineral particles obtained from the High-Resolution Image and the Standard Image. In the figure, the blue bar chart represents the cumulative frequency of mineral particles in each particle size range for the High-Resolution Image, while the red bar chart corresponds to the statistical results of the Standard Image. Due to the difference in the total number of particles between the two, the trend of the red bar chart in the main graph is less pronounced. To facilitate visual comparison, a separate chart is added on the right side of Figure 11, displaying the cumulative frequency of particles in each size range for the Standard Image. The blue and red dotted line graphs in the figure represent the cumulative percentage of particles within different size ranges relative to the total number of particles in the High-Resolution Image and Standard Image, respectively.

The statistical results in Figure 11 show that there are a total of 588,513 identifiable mineral particles in the high-resolution image, approximately 34 times the total number of identifiable particles (17,428) in the Standard Image. In the particle size range greater than 0.075 mm, the differences in the number of particles in each size segment between the two images are small, and no significant differences in magnitude are observed, indicating that both the High-Resolution Image and the Standard Image can retain most of the macroscopic structural information and exhibit good consistency in this aspect. In contrast, in the particle size range smaller than 0.075 mm, the High-Resolution Image shows a significant advantage. In the statistical results of Figure 11, the High-Resolution Image contains the largest number of identifiable particles in the 16–8 μm size range, with 239,046 particles, accounting for 40% of the total number of particles. In the Standard Image, this particle size range accounts for only 5% of the total (890 particles), which is significantly smaller in both quantity and proportion compared to the High-Resolution Image. In the Standard Image, the size range with the largest number of identifiable particles is 0.075–0.0325 mm, with 4147 particles, accounting for 24% of the total. In the High-Resolution Image, the number of particles in the same size range is 19,117, accounting for only 5% of the total. For particles in the mesoscopic scale range (less than 0.075 mm), identifiable particles in the Standard Image account for only 21% of the total, while in the High-Resolution Image, this number reaches 95%, showing a significant difference in the ability to capture mesoscopic structural information between the two images. The significant divergence in the cumulative distribution percentage curves of the two types of images in Figure 11 begins at the mesoscopic scale (below the 0.075 mm particle size range), further confirming the significant improvement in the fidelity of microstructural details in the High-Resolution Image. The High-Resolution Image exhibits a magnitude advantage in the number of identifiable particles at the mesoscopic scale, fully demonstrating its outstanding performance in preserving mesoscopic structural information.

In summary, the frequency distribution and cumulative percentage results shown in Figure 11 indicate that the high-resolution image acquisition and processing method proposed in this study not only accurately identifies mineral particles with a particle size ≥0.075 mm, but also demonstrates exceptional sensitivity and restoration ability in the identification of mesoscopic particles with a particle size <0.075 mm. This method significantly enhances the image’s ability to capture information at the mesoscopic scale, fully preserving the morphology and boundary features of mesoscopic mineral particles. As a result, it provides reliable data support for the high-fidelity digitalization of the internal meso-structure of asphalt mixtures and serves as a strong basis for multi-scale coupling modeling.

### 5.2. Accuracy Verification

In this study, *categorical cross-entropy* [62] (subjective matching cross-entropy) is employed to quantify the difference between the model’s sampling output and the ground truth from a probabilistic perspective, as defined in Equation (2). $H\left(p,q\right)$ serves as an evaluation parameter that reflects the divergence between the model-generated sampling results and the provided training samples.

(3) $$H\left(p,q\right)=-\sum_{i=1}^{n}p\left(x_{i}\right)logq\left(x_{i}\right)$$

p(x)—manually annotated training samples;

q(x)—the sampling results produced by the trained model;

H(p,q)—subjective matching cross-entropy, H(p,q)∈(0,1). A smaller cross-entropy indicates a higher degree of similarity between the two distributions, while a larger value indicates a lower degree of similarity.

Based on the trained U-Net model, a comparison of the segmentation performance between the High-Resolution Image and the Standard Image reveals that the former demonstrates a significant advantage in aggregate particle recognition accuracy. The segmentation accuracy of the High-Resolution Image reaches 99.3%, notably higher than the 98.1% of the Standard Image. The difference in values between the two clearly proves the high accuracy of the information contained in the High-Resolution Image, which holds substantial significance in large-scale image semantic segmentation tasks. This result indicates that the High-Resolution Image provides a more complete and more high-fidelity mineral boundary and morphological features for deep neural networks, especially in regions with smaller particle sizes, blurred contours, or complex particle boundaries, where fine structures are more accurately captured and segmented by the network. This effectively enhances the model’s generalization ability and local region interpretation accuracy. This difference not only highlights the profound impact of image resolution on the model’s perception capability but also further validates the significant value of high-resolution image acquisition and processing methods in the meso-structural numerical modeling and reconstruction of asphalt mixtures.

## 6. Conclusions

This paper presents a sub-pixel precision image acquisition and processing method designed for observing the mortar distribution within RAP–asphalt mixtures and asphalt film structures. The sub-pixel images obtained through this method provide a reliable basis for advancing the study of RAP mixture performance and underlying mechanisms. This paper introduces a computer-aided sub-pixel precision image acquisition method, which uses a digital camera as the core imaging device, focuses on focus stacking and texture suppression as the primary image processing techniques, and employs the deep neural network U-Net as the image segmentation architecture. This method effectively enhances image clarity and structural integrity, achieving high-resolution segmentation of aggregate particles in asphalt mixture cross-sectional images. It is a method capable of sub-pixel precision image acquisition and processing. The method is systematic and easy to operate and exhibits good repeatability and scalability. It is not only applicable to the study of meso-structures in asphalt mixtures but can also be extended to the digital analysis of meso-structural features in other composite materials, precision components, and mesoscopic structures.

The experiments demonstrated that compared with traditional standard images, the High-Resolution Image obtained using this method shows significant advantages in detail restoration and structural information retention. In the U-Net segmentation results, the High-Resolution Image achieved a segmentation accuracy of 99.3%, which is superior to the 98.1% of the Standard Image. Additionally, it significantly improves the recognition ability of mesoscopic particles (<0.075 mm), with the total number of particles identified being approximately 34 times that of the Standard Image, greatly enhancing the perception and reconstruction capability of mesoscopic particle structures at sub-pixel precision. Further statistical analysis also indicates that the quantity of fine particles and their proportion in the total particle count in the High-Resolution Image better match the actual particle gradation distribution of asphalt mixtures, thus providing a solid data foundation for multiscale coupling modeling of internal structures and micro–meso–macro performance analysis of asphalt mixtures.

In conclusion, the integrated image acquisition–processing–segmentation technology system developed in this paper not only provides a reliable pathway for high-resolution digital image research of asphalt mixtures but also establishes a practical framework for analyzing the mortar distribution within RAP mixtures and the characteristics of asphalt film structures. By enabling accurate recognition of these mesoscopic features, the method contributes to a deeper understanding of the mechanisms governing recycled asphalt performance. At the same time, it offers a general reference for other engineering and material studies that require image reconstruction and structural analysis at the mesoscopic scale. Building upon this technology, more authentic and reliable digital images of asphalt mixtures can be obtained, thereby providing a solid foundation for image-based reconstruction and simulation of digital models of asphalt mixtures. Reliable numerical models of asphalt mixtures are essential for accurate mechanical simulations in practical engineering. This study not only enhances the precision of image acquisition for asphalt mixtures, but also advances computer-aided numerical modeling, mechanical simulation, and performance analysis in real-world engineering applications. Looking ahead, this approach could be further applied to areas such as crack identification, material defect detection, and other high-resolution image analysis tasks, demonstrating both academic value and broad engineering application potential.

## 7. Outlook

The experimental method described in this paper focuses on the smooth cross-section of asphalt mixtures as the target for collection and provides the corresponding sample preparation requirements. To minimize the melting of temperature-sensitive asphalt and prevent damage to the cross-sectional structure during cutting, this method utilizes a water-cooled cutting process. Water-cooled cutting is currently the most commonly used method for cutting asphalt mixtures and causes the least disturbance to the surface structure of the mixture. Under these sample preparation conditions, the microscopic structural features of the smooth cross-section are subject to the greatest disturbance, while the mesoscopic structural features, which are the focus of this method, experience relatively less interference. In this case, although disturbances occur, they are consistent as long as the cutting conditions remain the same. This experimental method has not identified a cutting method that generates no heat, and future research is expected to improve in this direction. The high cost of the high-resolution imaging equipment required in this method remains a major challenge for its widespread adoption. Future advances in visual sensing technology and its large-scale application are expected to significantly reduce hardware costs, thereby enhancing the feasibility of this technology for practical engineering.

## Figures and Tables

**Table 1:** **Gradation of RAP mixtures.**

**Sieve Size (mm)**	13.2	9.5	4.75	2.36	1.18	0.6	0.3	0.15	0.075
**Original** **Passing Rate (%)**	96.2	91.1	14.3	1.4	0.7	0.5	0.4	0.3	0.1
**Pure Aggregate** **Passing Rate (%)**	100	99.2	42.9	25.3	19.5	14.1	10.5	8.7	4.8

**Table 2:** **Gradation of asphalt mixture specimens.**

**Sieve Size (mm)**	26.5	19	16	13.2	9.5	4.75	2.36	1.18	0.6	0.3	0.15	0.075
**Gradation**	100.0	88.9	77.7	64.4	48.5	41.3	30.5	22.2	15.8	9.7	7.1	5.6

**Table 3:** **Fundamental properties of asphalt binder.**

Test Item		Value
Penetration (25 °C, 100 g, 5 s), 0.1 mm		68.7
Softening Point (Ring and Ball Method), °C		52.3
Viscosity at 135 °C, Pa·s		0.425
Solubility, %		99.7
Flash Point (Cleveland Open Cup), °C		277
Density, g/cm^3^		1.022
Ductility (5 cm/min, 15 °C), cm		>150
Thin Film Oven Test (163 °C, 5 h)	Mass Loss, %	0.2
	Retained Penetration Ratio, %	77
	Ductility at 15 °C, cm	59

**Table 4:** **Fundamental properties of asphalt mixture.**

Residual Binder-to-Aggregate Ratio (%)	Theoretical Maximum Relative Density	Bulk Specific Gravity	Air Voids (%)	Oil Ratio (%)
4.2	2.492	2.410	3.3	4.3

## Data Availability

The original contributions presented in this study are included in the article material. Further inquiries can be directed to the corresponding author.
